# Number-needed-to-treat analysis of clinical progression in patients with metastatic castration-resistant prostate cancer in the STRIVE and TERRAIN trials

**DOI:** 10.1186/s12894-018-0387-7

**Published:** 2018-09-06

**Authors:** Neil M. Schultz, Neal D. Shore, Simon Chowdhury, Laurence H. Klotz, Raoul S. Concepcion, David F. Penson, Lawrence I. Karsh, Hongbo Yang, Bruce A. Brown, Arie Barlev, Scott C. Flanders

**Affiliations:** 1 Astellas Pharma, Inc., 1 Astellas Way, Northbrook, IL 60062 USA; 2 Carolina Urologic Research Center, Myrtle Beach, SC USA; 3 Guy’s, King’s, and St. Thomas’ Hospitals, London, UK; 4 Sunnybrook Health Sciences Centre, University of Toronto, Toronto, ON Canada; 5 Urology Associates, P.C, Nashville, TN USA; 6 Vanderbilt University Medical Center, Nashville, TN USA; 7 The Urology Center of Colorado, Denver, CO USA; 8 Analysis Group, Inc., Boston, MA USA; 9 Medivation, Inc., San Francisco, CA USA; 10 Pfizer, Inc., New York, NY USA

**Keywords:** Enzalutamide, Bicalutamide, Metastatic castration-resistant prostate cancer, Number needed to treat

## Abstract

**Background:**

This analysis estimated the number needed to treat with enzalutamide versus bicalutamide to achieve one additional patient with chemotherapy-naïve metastatic castration-resistant prostate cancer who would obtain clinical benefit regarding progression-free survival, radiographic progression-free survival, or no prostate-specific antigen progression at 1 and 2 years following treatment initiation.

**Methods:**

Clinical event rates were obtained from the STRIVE (NCT01664923) and TERRAIN (NCT01288911) trials, and the number needed to treat was the inverse of the absolute rate difference between the event rates of enzalutamide and bicalutamide. The 95% Confidence Interval of the number needed to treat was derived from the 95% Confidence Interval of the event rate difference.

**Results:**

Using STRIVE data (patients with metastatic disease: *n* = 128 enzalutamide; *n* = 129 bicalutamide) comparing enzalutamide with bicalutamide at 1 and 2 years, the numbers needed to treat to achieve one additional patient with chemotherapy-naïve metastatic castration-resistant prostate cancer with progression-free survival were 2.0 and 2.8, respectively; with radiographic progression-free survival, 2.6 and 3.0, respectively; and without prostate-specific antigen progression, 1.8 and 2.4, respectively. Using TERRAIN data (*n* = 184 enzalutamide; *n* = 191 bicalutamide) comparing enzalutamide with bicalutamide at 1 and 2 years, the numbers needed to treat to achieve one additional patient with progression-free survival were 4.3 and 3.7, respectively; with radiographic progression-free survival, 10.0 and 2.8, respectively; and without prostate-specific antigen progression, 2.1 and 3.2, respectively.

**Conclusions:**

The combined data from TERRAIN and STRIVE demonstrated that treating chemotherapy-naïve metastatic castration-resistant prostate cancer with enzalutamide leads to more patients without clinical progression at 1 and 2 years than with bicalutamide.

**Trial registration:**

STRIVE (NCT01664923; registration date: August 10, 2012) and TERRAIN (NCT01288911; registration date: February 1, 2011).

## Background

Prostate cancer (PC) is the second leading cause of cancer-related deaths and the most commonly diagnosed cancer among men worldwide [1, 2]. Castration-resistant prostate cancer (CRPC) is characterized by a castrate level of testosterone and either rising prostate-specific antigen (PSA) or radiographic disease progression [3]. CRPC may account for approximately 10–20% of PC cases, with over 84% of these cases demonstrating radiographic findings of metastatic CRPC (mCRPC) [4].

Until 2010, treatment for mCRPC was largely limited to taxane chemotherapy (docetaxel) or the oral non-steroidal antiandrogen bicalutamide plus luteinizing hormone-releasing hormone (LHRH) analogs [5]. Bicalutamide is a partial androgen receptor (AR) antagonist approved by the United States Food and Drug Administration (FDA) in 1995 as a 50 mg daily tablet for the treatment of metastatic androgen-sensitive PC, in combination with an LHRH analog [5, 6]. However, bicalutamide has been frequently used to treat various stages of mCRPC as monotherapy or as combination therapy with androgen-deprivation therapy despite a void of Category 1 evidence for its use in this patient population [7]. Until recently, median overall survival, depending on symptomatology and tumor burden, was estimated to be 9–18 months for those with mCRPC [4]. However, since 2010, the approval of new treatments for mCRPC has resulted in increases in median overall survival ranging from 16 to 35 months [7, 8].

One of these new therapies is the AR antagonist enzalutamide (Xtandi®; Astellas Pharma, Inc., IL, and Medivation, Inc., CA, which was acquired by Pfizer, Inc. in September 2016), which was approved by the FDA in 2012 [9]. Enzalutamide, with an approved dose of 160 mg daily for the treatment of mCRPC [9], is shown to have a five- to eight-fold higher AR binding affinity compared to bicalutamide in a preclinical test [10]. Enzalutamide targets three aspects of the AR signaling pathway: blocking androgen binding to ARs; inhibiting nuclear translocation of ARs; and inhibiting binding of ARs to DNA [11]. In contrast to bicalutamide, enzalutamide has received a Category 1 evidence recommendation for mCRPC in multiple US clinical guidelines [7, 12, 13].

Enzalutamide and bicalutamide have been directly compared in patients with chemotherapy-naïve CRPC in two randomized clinical trials: STRIVE and TERRAIN [14, 15]. In TERRAIN, enzalutamide and bicalutamide were compared in patients with chemotherapy-naïve asymptomatic or minimally symptomatic mCRPC [15]. The primary outcome of the TERRAIN trial was significantly improved progression-free survival (PFS) in patients receiving enzalutamide compared to patients receiving bicalutamide (15.7 months vs. 5.8 months, respectively). Median radiographic PFS (rPFS) was not reached for enzalutamide and was 16.4 months for bicalutamide, and median time to PSA progression was 19.4 months and 5.8 months, respectively. The STRIVE trial compared these two therapies in chemotherapy-naïve non-metastatic CRPC patients and mCRPC patients [14]. In patients with mCRPC, the trial reported a longer median PFS with enzalutamide than with bicalutamide (16.5 months vs. 5.5 months, respectively), and median rPFS was not reached with enzalutamide and was 8.3 months with bicalutamide. Median time to PSA progression was 24.9 months with enzalutamide and 5.7 months with bicalutamide. With respect to the incidence of serious adverse events, in the TERRAIN trial, patients treated with enzalutamide were more likely to experience a serious adverse event than patients treated with bicalutamide (31% vs. 23%, respectively); however, in the STRIVE trial, the rates were similar between the two treatment groups (29% vs. 28%, respectively) [14, 15].

The outcomes data from TERRAIN and STRIVE can be used to generate additional comparative efficacy evidence for enzalutamide versus bicalutamide that is applicable to mCRPC clinical practice and treatment decision-making. A useful and broadly used measure of treatment effect is the number needed to treat (NNT) to avoid a clinical progression event. The NNT is defined as the inverse of the absolute risk reduction [16] and reports the number of patients who need to be treated with one therapy versus an alternative therapy to achieve one additional clinical response or outcome. This approach has been previously used by the FDA to aid benefit-risk treatment comparisons [17] and is widely used in medical literature for its ease of interpretation. Thus, this analysis used outcomes data from the STRIVE and TERRAIN trials to calculate the NNT to avoid a clinical progression event (PFS, rPFS, or PSA progression) in patients with chemotherapy-naïve mCRPC receiving enzalutamide versus bicalutamide at 1 and 2 years.

## Methods

### Study population

This analysis reviewed data from patients with chemotherapy-naïve mCRPC in the STRIVE [14] (NCT01664923) and TERRAIN [15] (NCT01288911) trials. The definition of mCRPC was confirmed adenocarcinoma of the prostate, serum testosterone level less than 50 ng/dL, disease progression on androgen-deprivation therapy, and bone or soft tissue metastases in both trials [14, 15]. Approximately 65% of patients in the STRIVE trial had radiographic documentation of metastatic disease and all patients in the TERRAIN trial had mCRPC.

### Baseline patient characteristics

Baseline demographic categories of the study populations (age, race, weight, and body mass index) were reported. Clinical characteristics including Eastern Cooperative Oncology Group (ECOG) performance status score [18] and serum PSA levels were also summarized [14, 15].

### Clinical progression outcomes

Clinical progression events have been previously reported and included PFS, rPFS, and PSA progression at 1 and 2 years. These events were evaluated using end points defined in the STRIVE [14] and TERRAIN [15] trials that were similar but not identical (Table 1).

**Table 1:** Clinical outcome definitions used in the STRIVE and TERRAIN trials

Outcomes	STRIVE	TERRAIN
PFS	Time from randomization to the earliest objective evidence of PSA progression, radiographic disease progression, or death, whichever occurred first	Time from randomization to the first progression event (i.e. the earliest incidence of centrally determined radiographic disease progression, a skeletal-related event, or initiation of a new antineoplastic therapy) or death, whichever occurred first
rPFS	Time from randomization to the first objective evidence of radiographic disease progression or death, whichever occurred first	Time from randomization to the first objective evidence of radiographic disease progression or death, whichever occurred first. Radiographic progression in bone at or after Week 13 required a confirmatory bone scan
Freedom from PSA progression	Time from randomization to the earliest evidence of PSA progression, as per PCWG2 guidelines. PSA progression was defined as a > 25% increase in PSA with an absolute increase of > 2 ng/mL above the nadir	Time from randomization to the earliest evidence of a confirmed PSA progression, as per PCWG2 guidelines. PSA progression needs to be confirmed by a second consecutive value obtained ≥3 weeks later

*PCWG2* Prostate Cancer Working Group 2, *PFS* progression-free survival, *PSA* prostate-specific antigen, *rPFS* radiographic progression-free survival

### NNT analysis

The NNT to achieve one additional patient free from clinical progression was calculated as the reciprocal of the rate difference between enzalutamide and bicalutamide at 1 and 2 years. The 95% Confidence Interval (CI) of the NNT was calculated as the inverse of the 95% CI of the rate difference when comparing enzalutamide with bicalutamide. In this analysis, the NNT value represents the number of patients who needed to be treated with enzalutamide versus bicalutamide to achieve one additional patient with PFS, rPFS, or no PSA progression. Lower NNT values indicate greater benefit of enzalutamide over bicalutamide. The time points at 1 and 2 years were selected because the median follow-up was 17 months for both the enzalutamide and bicalutamide arms in the STRIVE trial and 20 and 17 months for the enzalutamide and bicalutamide arms, respectively, in the TERRAIN trial. In the TERRAIN trial at 2 years, the median time to events was reached for the majority of the evaluated clinical progression events (except rPFS).

NNT analyses were conducted separately from the STRIVE and TERRAIN trial data. For STRIVE, one- and two-year rates and standard errors of PFS, rPFS, and freedom from PSA progression with enzalutamide and bicalutamide were derived from the available clinical study report [11]. For TERRAIN, one- and two-year rates of PFS, rPFS, and no PSA progression with enzalutamide and bicalutamide were derived from the digitized Kaplan-Meier curves. Pseudo-individual patient data were generated from the digitized Kaplan-Meier curves according to the algorithm described in Guyot et al. [15, 19] were used to estimate the standard errors of the one- and two-year rates of the end point outcomes using Greenwood’s formula [20]. The 95% CIs of the rate difference were estimated based on the point estimate and standard error of each individual rate.

## Results

### Baseline characteristics

A total of 257 (128 enzalutamide, 129 bicalutamide) patients with chemotherapy-naïve mCRPC in STRIVE [14] and 375 (184 enzalutamide, 191 bicalutamide) patients in TERRAIN [15] were included in the analysis. At baseline, the median ages of the STRIVE patients receiving enzalutamide or bicalutamide were 71 and 72 years, respectively; in TERRAIN, the median age of patients receiving either treatment was 71 years. The majority of patients in both trials were white. The distribution of ECOG scores was similar in both trials for patients receiving enzalutamide or bicalutamide, and median serum PSA levels were higher in TERRAIN than in STRIVE (Table 2).

**Table 2:** Baseline characteristics of chemotherapy-naïve mCRPC patients in the STRIVE and TERRAIN trials

Baseline characteristics	STRIVE		TERRAIN	
	Enzalutamide (*n* = 128)	Bicalutamide (*n* = 129)	Enzalutamide (*n* = 184)	Bicalutamide (*n* = 191)
Age, years				
Median	71	72	71	71
Range	46–87	50–90	50–96	48–91
Race, *n* (%)				
Black or African-American	14 (10.9)	15 (11.6)	8 (4.3)	10 (5.2)
White	107 (83.6)	111 (86.0)	172 (93.4)	176 (92.1)
Other	7 (5.5)	3 (2.3)	4 (2.2)	5 (2.6)
Baseline weight, kg				
Median	91.6	88.3	88.2	86.8
Range	58.5–166.6	52.7–181.8	57.0–184.1	56.0–143.5
Body mass index, kg/m^2^				
Median	30	29	28	28
Range	20–49	16–62	18–51	18–44
ECOG performance status, *n* (%)				
0	92 (71.9)	92 (71.3)	130 (70.7)	146 (76.4)
1	36 (28.1)	37 (28.7)	54 (29.3)	45 (23.6)
Serum PSA, μg/L				
Median	15.1	18.3	21	22
Range	0.0–1499.7	0.2–2849.7	0.6–5000	0.1–4681

*ECOG* Eastern Cooperative Oncology Group, *mCRPC* metastatic castration-resistant prostate cancer, *PSA* prostate-specific antigen

### Rates of clinical progression in STRIVE

At 1 year, PFS rates were 59.5% for enzalutamide patients and 9.4% for bicalutamide patients in STRIVE, a difference of 50.1% (95% CI 39.5–60.7) [Table 3]. rPFS rates at 1 year were 74.0 and 35.1% for enzalutamide and bicalutamide patients, respectively, a difference of 38.9% (95% CI 24.7–53.1). At 1 year, 69.4 and 12.9% of enzalutamide and bicalutamide patients, respectively, were free from PSA progression, a difference of 56.5% (95% CI 45.4–67.7).

**Table 3:** Rates of PFS, rPFS, and freedom from PSA progression in the STRIVE and TERRAIN trials

Outcome	STRIVE			TERRAIN		
	Enzalutamide, %	Bicalutamide, %	Enzalutamide versus bicalutamide difference, % (95% CI)	Enzalutamide, %	Bicalutamide, %	Enzalutamide versus bicalutamide difference, % (95% CI)
PFS						
One year	59.5	9.4	50.1 (39.5–60.7)	55.0	31.7	23.3 (12.6–34.0)
Two years	40.0	4.1	35.9 (23.4–48.4)	37.8	11.0	26.8 (15.0–38.7)
rPFS						
One year	74.0	35.1	38.9 (24.7–53.1)	68.2	58.1	10.1 (−2.6–22.7)
Two years	56.4	23.2	33.1 (13.9–52.4)	61.3	25.6	35.7 (18.3–53.0)
Freedom from PSA progression						
One year	69.4	12.9	56.5 (45.4–67.7)	65.1	18.6	46.5 (34.6–58.5)
Two years	50.3	8.1	42.2 (27.0–57.4)	41.3	10.3	31.0 (16.9–45.1)

*CI* Confidence Interval, *PFS* progression-free survival, *PSA* prostate-specific antigen, *rPFS* radiographic progression-free survival

Similar to the 1-year results in STRIVE, enzalutamide resulted in superior outcomes relative to bicalutamide at 2 years (Table 3). At 2 years, PFS rates were 40.0% for enzalutamide patients and 4.1% for bicalutamide patients, a difference of 35.9% (95% CI 23.4–48.4). rPFS rates at 2 years were 56.4 and 23.2% for enzalutamide and bicalutamide patients, respectively, a difference of 33.1% (95% CI 13.9–52.4). At 2 years, 50.3 and 8.1% of enzalutamide and bicalutamide patients, respectively, were free from PSA progression, a difference of 42.2% (95% CI 27.0–57.4).

### NNT in STRIVE

**Fig. 1:**
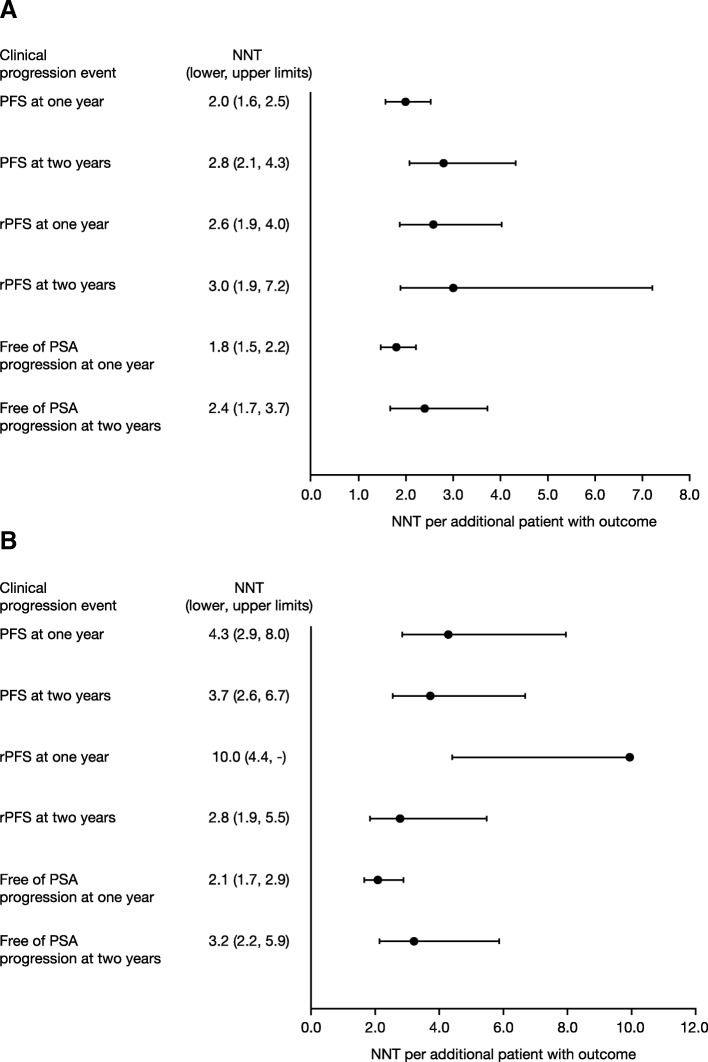
NNT for PFS, rPFS, and freedom from PSA progression comparing enzalutamide with bicalutamide. NNT for the STRIVE (**a**) and TERRAIN (**b**) trials at 1 and 2 years. The lower limit of NNT for rPFS at 1 year was not reported, as the rate difference between enzalutamide with bicalutamide covers the 0, and it is not meaningful to report a negative NNT value. *NNT* number needed to treat, *PFS* progression-free survival, *PSA* prostate-specific antigen, *rPFS* radiographic progression-free survival

For STRIVE, the NNT for PFS was 2.0 (upper, lower limits: 1.6, 2.5) when comparing enzalutamide and bicalutamide; thus, treating two patients with enzalutamide resulted in one additional patient with PFS at 1 year versus treating with bicalutamide (Fig. 1). At 2 years, the NNT for PFS was 2.8 (upper, lower limits: 2.1, 4.3) when comparing enzalutamide and bicalutamide. The NNT for rPFS at 2 year was 2.6 (upper, lower limits: 1.9, 4.0) and at 2 years was 3.0 (1.9, 7.2) when comparing enzalutamide and bicalutamide. Lastly, the NNT for freedom from PSA progression was 1.8 (upper, lower limits: 1.5, 2.2) at 1 year and 2.4 (1.7, 3.7) at 2 years when comparing enzalutamide and bicalutamide.

### Rates of clinical progression in TERRAIN

At 1 year, PFS rates were 55.0% for enzalutamide patients and 31.7% for bicalutamide patients in TERRAIN, a difference of 23.3% (95% CI 12.6–34.0) [Table 3]. rPFS rates at 1 year were 68.2 and 58.1% for enzalutamide and bicalutamide patients, respectively, a difference of 10.1% (95% CI − 2.6-22.7). At 1 year, 65.1 and 18.6% of enzalutamide and bicalutamide patients, respectively, were free from PSA progression, a difference of 46.5% (95% CI 34.6–58.5).

Similar to the one-year results in TERRAIN, enzalutamide also resulted in superior outcomes relative to bicalutamide at 2 years (Table 3). At 2 years, PFS rates were 37.8% for enzalutamide patients and 11.0% for bicalutamide patients, a difference of 26.8% (95% CI 15.0–38.7). rPFS rates at 2 years were 61.3 and 25.6% for enzalutamide and bicalutamide patients, respectively, a difference of 35.7% (95% CI 18.3–53.0). Lastly, 41.3% and 10.3% of enzalutamide and bicalutamide patients, respectively, were free from PSA progression at 2 years, a difference of 31.0% (95% CI 16.9–45.1).

### NNT in TERRAIN

For TERRAIN, the NNT for PFS at 1 year was 4.3 (upper, lower limits: 2.9, 8.0) and at 2 years was 3.7 (2.6, 6.7) when comparing enzalutamide with bicalutamide (Fig. 1). The NNT for rPFS at 1 year was 10.0 (upper, lower limits: 4.4, not evaluable) and at 2 years was 2.8 (1.9, 5.5) when comparing enzalutamide with bicalutamide. The NNT for freedom from PSA progression was 2.1 (upper, lower limits: 1.7, 2.9) at 1 year and 3.2 (2.2, 5.9) at 2 years when comparing enzalutamide with bicalutamide.

## Discussion

This analysis used data from the STRIVE [14] and TERRAIN [15] clinical trials comparing two AR inhibitor therapies – enzalutamide and bicalutamide – for the treatment of chemotherapy-naïve patients with mCRPC to calculate NNT to avoid clinical progression. The outcomes reported in the trials were translated into NNTs, a metric of comparative efficacy that can be utilized to inform decision-making in clinical practice. The results show that at 1 year, the NNTs when comparing enzalutamide with bicalutamide in STRIVE and TERRAIN were 2.0 and 4.3, respectively for PFS; 2.6 and 10.0, respectively, for rPFS; and 1.8 and 2.1, respectively, for no PSA progression. At 2 years, the NNTs comparing enzalutamide with bicalutamide in STRIVE and TERRAIN were 2.8 and 3.7, respectively, for PFS; 3.0 and 2.8, respectively, for rPFS; and 2.4 and 3.2, respectively, for no PSA progression.

It is important to consider that NNT values estimated from the STRIVE and TERRAIN trials are generally consistent across time points and across the different clinical trial populations, with the exception of rPFS at 1 year. The NNT of rPFS at 1 year estimated from the TERRAIN trial was 10.0 and the CI of the rPFS rate difference crossed 0; however, the respective NNT value estimated from the STRIVE trial was 2.6. At 2 years, the NNTs for rPFS were similar across the trials, with the values estimated at 3.0 (upper, lower limits: 1.9, 7.2) and 2.8 (upper, lower limits: 1.9, 5.5) for STRIVE and TERRAIN, respectively. This difference indicates that the uncertainties associated with the benefit of enzalutamide versus bicalutamide decreased over the long term. Thus, the numerically lower NNT values for enzalutamide versus those for bicalutamide demonstrate that enzalutamide for mCRPC leads to more patients free from disease progression or death (i.e. PFS), radiographic disease progression, and PSA progression compared with bicalutamide at 1 and 2 years.

NNT analysis was selected to compare enzalutamide with bicalutamide for the treatment of mCRPC because it is an established and interpretable measure that can be used in clinical practice to illustrate treatment effectiveness. This approach has been previously applied in evaluating treatments in PC [21, 22]. For example, Massoudi et al. [22] compared enzalutamide with abiraterone plus prednisone using data from the PREVAIL [23] and COU-AA-302 [24, 25] clinical trials. They reported an NNT of 14 for rPFS, indicating that treating 14 patients with enzalutamide instead of abiraterone plus prednisone would yield one extra patient free of radiographic progression or death at 1 year. When comparing therapies on efficacy outcomes, in general, lower NNTs indicate treatment superiority. The smallest possible NNT is 1; a value that translates for every patient treated with a therapy, there would be a benefit that would not be reached with the comparative treatment. However, values can range widely across NNT comparisons and there is no established threshold for an NNT value to be considered clinically meaningful.

Hildebrandt et al. [26] conducted a literature review of the use of NNT calculations alongside randomized controlled trials (2003 to 2005) and noted that 62 of 734 eligible trials reported NNTs with values ranging from 2 to 325.7. Therefore, each individual NNT measure needs to be evaluated for its clinical interpretation based on the disease and outcomes used for the evaluation. That being said, the value of NNT is evident based on the emerging number of recent publications using this methodology when evaluating oncology treatment options [27–31]. In addition, the 2018 National Comprehensive Cancer Network (NCCN) guidelines for prostate cancer cite studies that report NNT estimates in the discussion of active surveillance and radical prostatectomy [7]. Furthermore, the NNT methodology provides a transparent interpretation of the relative risk for a particular outcome that can be utilized in clinical practice decision-making.

The value of the present analysis is to translate the statistically significant outcome rate differences reported in STRIVE and TERRAIN into a clear effect-size measure relevant to real-world clinical practice and treatment choice when considering enzalutamide versus bicalutamide. With the exception of the NNT for rPFS at 1 year in TERRAIN, all of the NNTs were similar and demonstrated a robust effect size and clinical benefit of enzalutamide over bicalutamide and overall agreement among the robust Phase II trials.

Historically, bicalutamide has been commonly used in the treatment of various stages of PC due to its global accessibility, relatively low cost, once-daily dosing formulation, well-established safety profile, and ability to reduce PSA levels [5, 32, 33]. Bicalutamide can be used as monotherapy or combination therapy (approved in the United States at 50 mg once daily), and its efficacy in PC has been reported in several studies. For example, a 1996 randomized, double-blind, multicenter study compared 50 mg once-daily bicalutamide plus LHRH with 250 mg (3 times a day) flutamide plus LHRH in patients with untreated metastatic PC and reported that bicalutamide was better tolerated than flutamide, although efficacy was similar [33]. Additionally, Klotz et al. [32] reported a 20% reduction in risk of death in metastatic PC patients receiving 50 mg once-daily bicalutamide compared with castration alone.

However, the recent availability of several new therapies for chemotherapy-naïve mCRPC presents opportunities for physicians and patients to optimize treatment decision-making in consideration of all approved therapeutic options and current association guidelines. In particular, among the FDA-approved treatments for chemotherapy-naïve mCRPC, enzalutamide has received recommendations in the NCCN guidelines based on Category 1 evidence, and these recommendations have been adopted in clinical practice [7, 34]. The pivotal trial of enzalutamide (PREVAIL) [23] compared the drug with placebo among chemotherapy-naïve patients and observed improved overall survival (median survival of 32.4 months vs. 30.2 months, respectively) and rPFS (65% vs. 14% at 12 months). The AFFIRM trial [35] assessed patients previously treated with docetaxel-based chemotherapy and also found improved overall survival for enzalutamide versus placebo (median survival of 18.4 months vs. 13.6 months, respectively) and improved rPFS (median of 8.3 months vs. 2.9 months, respectively). As currently discussed, both STRIVE and TERRAIN reinforced the superiority of enzalutamide over bicalutamide for chemotherapy-naïve mCRPC [14, 15]. In addition, in comparison with the PREVAIL and AFFIRM trials which allowed progression on previous bicalutamide, progression on prior bicalutamide was not allowed in the STRIVE and TERRAIN trials [14, 15, 23, 35]. Therefore, these four clinical trials showed clinical efficacy for enzalutamide among patient populations with diverse treatment history (e.g. chemotherapy-naïve, post chemotherapy, bicalutamide-naïve, and bicalutamide-experienced).

The availability of evidence from STRIVE and TERRAIN, as well as this NNT comparison, help establish optimal treatment strategies for mCRPC and may result in a change in the use of enzalutamide and bicalutamide in clinical practice. Future studies could use similar NNT methodology to indirectly compare enzalutamide with other existing and emerging hormonal therapies for the treatment of chemotherapy-naïve mCRPC. In addition, NNT analyses based on follow-up data beyond 2 years from STRIVE and TERRAIN would provide additional value.

### Limitations

In addition to the previous comments regarding NNT analyses, this research is subject to the following limitations. First, patients enrolled in clinical trials might not be representative of the overall mCRPC population in real-world clinical practice. Trial-specific events and case report forms may assess outcomes more rigorously than real-world practice. Second, overall survival was not evaluated in the current analysis because the STRIVE and TERRAIN trials did not include it as a standalone end point; therefore the overall survival rate was not reported in the publications [14, 15]. Third, in this analysis, evaluations of clinical progression outcomes were limited to 1 and 2 years due to the availability of the data; however, NNT can be evaluated at additional time points. Fourth, the current NNT analysis focused on clinical efficacy; NNTs related to safety or quality-of-life outcomes were not examined, although future studies evaluating this topic would be valuable. Lastly, while this study focused on NNT comparing these two treatments, future studies should also consider evaluating the cost and cost-efficacy of these two therapies in the mCRPC population.

## Conclusions

The results from the current NNT analysis of chemotherapy-naïve mCRPC patients in STRIVE and TERRAIN indicate that treatment with enzalutamide will lead to more patients free from disease progression or death (i.e. PFS), radiographic disease progression, and PSA progression compared with bicalutamide at 1 and 2 years. In addition to the results of STRIVE and TERRAIN, this analysis may assist physicians and patients in choosing the optimal treatment for mCRPC.

## Abbreviations

AR: Androgen receptor
CI: Confidence Interval
CRPC: Castration-resistant prostate cancer
ECOG: Eastern Cooperative Oncology Group
FDA: United States Food and Drug Administration
LHRH: Luteinizing hormone-releasing hormone
mCRPC: Metastatic castration-resistant prostate cancer
NCCN: National Comprehensive Cancer Network
NNT: Number needed to treat
PC: Prostate cancer
PFS: Progression-free survival
PSA: Prostate-specific antigen
rPFS: Radiographic progression-free survival

## References

[1] Ferlay J, Soerjomataram I, Ervik M, Dikshit R, Eser S, Mathers C (2014). GLOBOCAN 2012 v1.0, Cancer Incidence and Mortality Worldwide: IARC CancerBase No. 11.

[2] Siegel RL, Miller KD, Jemal A (2016). Cancer statistics, 2016. CA Cancer J Clin.

[3] Cornford P, Bellmunt J, Bolla M, Briers E, De Santis M, Gross T (2017). EAU-ESTRO-SIOG guidelines on prostate cancer. Part II: treatment of relapsing, metastatic, and castration-resistant prostate cancer. Eur Urol.

[4] Kirby M, Hirst C, Crawford ED (2011). Characterising the castration-resistant prostate cancer population: a systematic review. Int J Clin Pract.

[5] Schellhammer PF, Sharifi R, Block NL, Soloway MS, Venner PM, Patterson AL (1997). Clinical benefits of bicalutamide compared with flutamide in combined androgen blockade for patients with advanced prostatic carcinoma: final report of a double-blind, randomized, multicenter trial. Urology.

[6] Food and Drug Administration (2009). Highlights of prescribing information: bicalutamide tablets.

[7] National Comprehensive Cancer Network (2018). NCCN clinical practice guidelines in oncology (NCCN guidelines®). Prostate Cancer.

[8] Roviello G, Sigala S, Sandhu S, Bonetta A, Cappelletti MR, Zanotti L (2016). Role of the novel generation of androgen receptor pathway targeted agents in the management of castration-resistant prostate cancer: a literature based meta-analysis of randomized trials. Eur J Cancer.

[9] Food and Drug Administration (2015). Xtandi [package insert].

[10] Rathkopf D, Scher HI (2013). Androgen receptor antagonists in castration-resistant prostate cancer. Cancer J.

[11] Schalken J, Fitzpatrick JM (2016). Enzalutamide: targeting the androgen signalling pathway in metastatic castration-resistant prostate cancer. BJU Int.

[12] Basch E, Loblaw DA, Oliver TK, Carducci M, Chen RC, Frame JN (2014). Systemic therapy in men with metastatic castration-resistant prostate cancer: American Society of Clinical Oncology and Cancer Care Ontario clinical practice guideline. J Clin Oncol.

[13] Cookson MS, Roth BJ, Dahm P, Engstrom C, Freedland SJ, Hussain M (2015). Castration-resistant prostate cancer: AUA guideline.

[14] Penson DF, Armstrong AJ, Concepcion R, Agarwal N, Olsson C, Karsh L (2016). Enzalutamide versus bicalutamide in castration-resistant prostate cancer: the STRIVE trial. J Clin Oncol.

[15] Shore N, Chowdhury S, Villers A, Klotz L, Siemens DR, Phung D (2016). Efficacy and safety of enzalutamide versus bicalutamide for patients with metastatic prostate cancer (TERRAIN): a randomised, double-blind, phase 2 study. Lancet Oncol.

[16] Laupacis A, Sackett DL, Roberts RS (1988). An assessment of clinically useful measures of the consequences of treatment. N Engl J Med.

[17] Center for Drug Evaluation and Research Office of Translational Sciences (2012). Manual of Policies and Procedures. Good Review Practice: Statistical Review Template.

[18] Oken MM, Creech RH, Tormey DC, Horton J, Davis TE, McFadden ET (1982). Toxicity and response criteria of the Eastern Cooperative Oncology Group. Am J Clin Oncol.

[19] Guyot P, Ades AE, Ouwens MJ, Welton NJ (2012). Enhanced secondary analysis of survival data: reconstructing the data from published Kaplan-Meier survival curves. BMC Med Res Methodol.

[20] Greenwood M (1926). A report on the natural duration of cancer.

[21] Dranitsaris G, Hatzimichael E (2012). Interpreting results from oncology clinical trials: a comparison of denosumab to zoledronic acid for the prevention of skeletal-related events in cancer patients. Support Care Cancer.

[22] Massoudi M, Balk M, Yang H, Bui CN, Pandya BJ, Guo J (2017). Number needed to treat and associated incremental costs of treatment with enzalutamide versus abiraterone acetate plus prednisone in chemotherapy-naïve patients with metastatic castration-resistant prostate cancer. J Med Econ.

[23] Beer TM, Armstrong AJ, Rathkopf DE, Loriot Y, Sternberg CN, Higano CS (2014). Enzalutamide in metastatic prostate cancer before chemotherapy. N Engl J Med.

[24] Ryan CJ, Smith MR, de Bono JS, Molina A, Logothetis CJ, de Souza P (2013). Abiraterone in metastatic prostate cancer without previous chemotherapy. N Engl J Med.

[25] Ryan CJ, Smith MR, Fizazi K, Saad F, Mulders PFA, Sternberg CN (2015). Abiraterone acetate plus prednisone versus placebo plus prednisone in chemotherapy-naive men with metastatic castration-resistant prostate cancer (COU-AA-302): final overall survival analysis of a randomised, double-blind, placebo-controlled phase 3 study. Lancet Oncol.

[26] Hildebrandt M, Vervölgyi E, Bender R (2009). Calculation of NNTs in RCTs with time-to-event outcomes: a literature review. BMC Med Res Methodol.

[27] Anderson D, Lehmann J, Ecker T, Vosgerau S, Donatz V (2017). Cost effectiveness of GnRH antagonists in patients with prostate cancer and cardiovascular risk: comparative analysis against Leuprorelin by the number needed to treat. Urologe A.

[28] de Carvalho TM, Heijnsdijk EAM, de Koning HJ (2016). Estimating the individual benefit of immediate treatment or active surveillance for prostate cancer after screen-detection in older (65+) men. Int J Cancer.

[29] Frandsen J, Orton A, Shrieve D, Tward J (2017). Risk of death from prostate cancer with and without definitive local therapy when Gleason pattern 5 is present: a surveillance, epidemiology, and end results analysis. Cureus.

[30] Ho R, Rufino C, Simões J, Alves M (2017). Number Needed to Treat (NNT) and Cost of Preventing an Event (COPE) comparison between the Association of Cobimetinib and Vemurafenib among other treatment options for metastatic melanoma with BRAF V600 mutation. Value Health.

[31] Löppenberg B, Dalela D, Karabon P, Sood A, Sammon JD, Meyer CP (2017). The impact of local treatment on overall survival in patients with metastatic prostate cancer on diagnosis: a national cancer data base analysis. Eur Urol.

[32] Klotz L, Schellhammer P, Carroll K (2004). A re-assessment of the role of combined androgen blockade for advanced prostate cancer. BJU Int.

[33] Schellhammer P, Sharifi R, Block N, Soloway M, Venner P, Patterson AL (1996). Maximal androgen blockade for patients with metastatic prostate cancer: outcome of a controlled trial of bicalutamide versus flutamide, each in combination with luteinizing hormone-releasing hormone analogue therapy. Urology.

[34] Ellis LA, Lafeuille MH, Gozalo L, Pilon D, Lefebvre P, McKenzie S (2015). Treatment sequences and pharmacy costs of 2 new therapies for metastatic castration-resistant prostate cancer. Am Health Drug Benefits.

[35] Scher HI, Fizazi K, Saad F, Taplin ME, Sternberg CN, Miller K (2012). Increased survival with enzalutamide in prostate cancer after chemotherapy. N Engl J Med.

