# Correction to: Number-needed-to-treat analysis of clinical progression in patients with metastatic castration-resistant prostate cancer in the STRIVE and TERRAIN trials

**DOI:** 10.1186/s12894-018-0397-5

**Published:** 2018-10-01

**Authors:** Neil M Schultz, Neal D Shore, Simon Chowdhury, Laurence H Klotz, Raoul S Concepcion, David F Penson, Lawrence I Karsh, Hongbo Yang, Bruce A Brown, Arie Barlev, Scott C Flanders

**Affiliations:** 1 Astellas Pharma, Inc., 1 Astellas Way, Northbrook, IL 60062 USA; 2 Carolina Urologic Research Center, Myrtle Beach, SC USA; 3 Guy’s, King’s, and St. Thomas’ Hospitals, London, UK; 4 Sunnybrook Health Sciences Centre, University of Toronto, Toronto, ON Canada; 5 Urology Associates, P.C, Nashville, TN USA; 6 Vanderbilt University Medical Center, Nashville, TN USA; 7 The Urology Center of Colorado, Denver, CO USA; 8 Analysis Group, Inc., Boston, MA USA; 9 Medivation, Inc., San Francisco, CA USA; 10 Pfizer, Inc., New York, NY USA

## Correction to Schultz et al. BMC Urology (2018) 18:77 DOI: 10.1186/s12894-018-0387-7

It has been highlighted that in the original article [1] there was a typesetting mistake in the Results – NNT in Strive section. This Correction article states the incorrect and correct sentence.

Incorrect:

The NNT for rPFS at **2** year was 2.6 (upper, lower limits: 1.9, 4.0) and at 2 years was 3.0 (1.9, 7.2) when comparing enzalutamide and bicalutamide.

Correct:

The NNT for rPFS at **1** year was 2.6 (upper, lower limits: 1.9, 4.0) and at 2 years was 3.0 (1.9, 7.2) when comparing enzalutamide and bicalutamide.
